# Immunological and periodontal benefits of prebiotic polydextrose in rats with induced periodontitis

**DOI:** 10.1002/jper.70095

**Published:** 2026-02-19

**Authors:** Raquel de S. F. Nassar, Marcella C. Ribeiro, Ana Carolina P. Hernandes, Laura Z. Gianduzzo, Vanessa de P. S. Pereira, Sérgio L. S. Salvador, Edilson Ervolino, Débora S. F. Sávio, Michel R. Messora, Flávia A. C. Furlaneto

**Affiliations:** ^1^ Department of Oral and Maxillofacial Surgery and Periodontology, Ribeirao Preto School of Dentistry University of Sao Paulo – USP Ribeirao Preto São Paulo Brazil; ^2^ Department of Clinical Analyses, School of Pharmaceutical Sciences of Ribeirao Preto University of Sao Paulo – USP Ribeirao Preto São Paulo Brazil; ^3^ Department of Basic Sciences São Paulo State University – UNESP, Araçatuba School of Dentistry Araçatuba São Paulo Brazil

**Keywords:** intestinal microbiome, periodontal disease, prebiotics

## Abstract

**Background:**

Prebiotic therapy is a promising approach for managing periodontitis due to its potential benefits in modulating the microbiome and the immune response. This study aimed to evaluate the effects of the prebiotic (PREB) polydextrose (PDX) on the development of experimental periodontitis (EP) in rats.

**Methods:**

A total of 44 male adult rats (*Rattus norvegicus, albinus*, Wistar) were randomly allocated into groups (*n* = 11) C (control), PREB, EP, and EP/PREB. The PREB and EP/PREB groups received 2 g of PDX/day in the drinking water for 44 days. The EP and EP/PREB groups received cotton ligatures around mandibular first molars (M1) for 14 days. Microtomographic, histopathological, and immunohistochemical analyses of periodontal and intestinal tissues were performed. Data were analyzed statistically (*p* < 0.05).

**Results:**

The EP/PREB group showed less alveolar bone destruction than the EP group. The EP/PREB group exhibited higher expressions of IL‐10 and a lower number of TRAP (tartrate‐resistant acid phosphatase)‐positive cells than the EP group in periodontal tissues. The EP/PREB group had increased villus heights in the duodenum and ileum, increased crypt depths in the duodenum, and higher expressions of occludin in all intestinal portions, claudin‐1 in the jejunum, IL‐10 in the duodenum, and lower expressions of IL‐1β in the jejunum compared with the EP group.

**Conclusion:**

Polydextrose exerted a protective effect against alveolar bone resorption and favorably modulated both local and systemic immune‐inflammatory response in rats with experimental periodontitis.

**Plain language summary:**

Prebiotic therapy has emerged as a promising approach for treating periodontitis, an immunoinflammatory disease associated with a dysbiotic biofilm that leads to the loss of the supporting structures of the teeth. This is because prebiotics can positively influence the balance of bacteria in the body and the immune system's response. However, current understanding of its potential effects and mechanisms of action remains limited in periodontal therapy. The effects of the prebiotic polydextrose (PDX) on periodontal tissues remain unknown. The results of the present study show that the prebiotic PDX can minimize alveolar bone loss and improve bone microarchitecture through modulation of the immune‐inflammatory response in both intestinal and periodontal tissues of rats with experimental periodontitis. Our findings suggest that the prebiotic PDX may represent a beneficial adjunctive strategy for managing periodontitis.

## 1 INTRODUCTION

Prebiotics (PREBs) are substrates that are selectively utilized by host microorganisms and confer health benefits.^1^ PREBs can be found naturally in certain foods,^2^ or can be synthetically produced and administered as supplements, in which they are usually present in higher concentrations.^3^ Studies have demonstrated the benefits of PREBs use in enhancing immune responses^4^ and alleviating symptoms of intestinal diseases,^5^ reducing the risk of cardiovascular diseases,^6^ non‐insulin‐dependent diabetes,^6^ and preventing bone‐related disorders such as osteopenia, and/or osteoporosis.^7^ PREB intake can increase the production of short‐chain fatty acids (SCFAs), namely acetic, butyric, and propionic acids, by the gut microbiota, which are widely known for their health‐promoting effects through various mechanisms, such as enhanced intestinal calcium absorption, reduced intestinal pH, and the promotion of intestinal villi development.^7^ Certain PREBs may favorably modulate the inflammatory response to pathogens and help to stabilize the intestinal mucosal barrier.^8^ In the field of periodontology, preclinical studies have identified potential PREBs capable of stimulating beneficial bacteria and suppressing pathogenic species in vitro,^9^ reducing alveolar bone loss,^10^ decreasing tumor necrosis factor (TNF)‐α and interleukin (IL)‐1β levels, and increasing transforming growth factor (TGF)‐β levels in gingival tissues of animals with experimental periodontitis (EP).^11^

Among the PREBs, polydextrose (PDX) has gained prominence due to its better tolerance compared with most other poorly digestible carbohydrates, a feature attributed to its high molecular weight and partial colonic fermentation.^12^ Animals receiving PDX exhibited increased immunoglobulin A (IgA) secretion in the cecum,^13^ along with enhanced absorption of calcium, magnesium,^14^ and iron.^15^ PDX consumption combined with a Western diet modulates the gut microbiota, leading to reductions in triglyceride and cholesterol levels, downregulation of genes that impair lipid metabolism (Dgat1, Cd36, and Fiaf), and upregulation of Fxr, a favorable regulator of lipid homeostasis in the intestinal tract of mice.^16^ In rats, supplementation with PDX and galactooligosaccharide increased the relative abundance of gut bacterial genera associated with health, and reduced fecal levels of deoxycholic acid and lithocholic acid, which are metabolites commonly linked to intestinal inflammation.^17^ In clinical studies, PDX has been shown to increase the production of SCFAs,^18^ reduce intestinal pH and postprandial triglyceride response,^18^, ^19^ and significantly improve bowel function,^20^ stool consistency,^20^ and satiety.^21^

Prebiotic therapy may represent a promising adjuvant strategy in periodontal treatment; however, knowledge regarding its potential effects and mechanisms of action is still in its infancy in dentistry.^9^ The effects of the PREB PDX on periodontal tissues remain unknown. Therefore, this study aims to evaluate the effects of the PREB PDX on the development of EP in rats.

## 2 MATERIALS AND METHODS

### 2.1 Sample

The ideal sample size to ensure 80% statistical power was calculated based on the differences in means and standard deviations between the EP and C groups in the study by Oliveira et al. (2017),^22^ recognizing a significant difference of 5% (δ) between groups, a 95% confidence interval (CI) (α = 0.05), a standard deviation (σ) of 23%, and changes in bone volume percentage assessed by micro‐computed tomography (BV/TV%) as the primary outcome variable. The *Z*‐score values used were

[*Z*α(1.96) + *Z*β(0.84)]^2 ^= 7.84. The sample size per group was calculated using the formula: $n\geq \left\{2\left[{\left(\sigma \right)}^{2}/{\left(\delta \right)}^{2}\right]\right\}\times {\left(Z\alpha +Z\beta \right)}^{2}$, resulting in a minimum requirement of eight animals per experimental group.^22^ Considering a possible 30% loss of animals and/or ligatures during the study, the sample consisted of 11 animals per experimental group. This study was conducted in accordance with the guidelines established by the National Council for the Control of Animal Experimentation (CONCEA), following the development of a research protocol that included the research question, experimental design, and statistical analysis plan, which was reviewed and approved by the Animal Use Ethics Committee (CEUA) of the School of Dentistry of Ribeirão Preto (FORP), University of São Paulo (USP) (Protocol No. 2021.1.351.58.2). The study is reported according to the ARRIVE statement.

### 2.2 Experimental model

A total of 44 male rats (*Rattus norvegicus*, *albinus*, Wistar), weighing between 300 and 320 g and aged between 3 and 4 months (Animal Facility, FORP/USP), were used. All animals were in good health and had not undergone any procedures before the experiment. They were housed at a controlled temperature of 22 ± 2°C with 12/12‐hour light–dark cycles. The animals were fed selected solid chow and had free access to water (ad libitum), and all procedures were performed at standardized times throughout the experiment. According to a numerical table generated using software, the animals were randomly assigned to four groups (*n* = 11): C (Control), EP, PREB (PREB PDX), and EP/PREB (PREB PDX + EP). Each group was housed in three cages (2 cages with 4 animals and 1 cage with 3 animals). No specific criteria for including or excluding animals or data points were established a priori, and all animals that met the general conditions of the study were included. No animals or data points were excluded from any experimental group.

### 2.3 Prebiotic therapy and periodontitis induction

From the beginning of the experiment (T–30) to its end (T+14), 2 g of polydextrose (PDX; Litesse Ultra, Danisco USA Inc., Terre Haute, IN, USA) was diluted in the drinking water of each animal in the PREB and EP/PREB groups once daily.^23^, ^24^, ^25^ Each animal was temporarily placed in an individual cage until the full consumption of its daily PDX dose. On day 0, cotton ligatures were placed around the right and left mandibular first molars (M1) of the animals in the EP and EP/PREB groups and kept in position for 14 days.^26^ To induce experimental periodontitis, the animals were anesthetized via intraperitoneal injection using a solution of 2% xylazine hydrochloride (2 mg/mL) (Rompum, Bayer Animal Health, São Paulo, SP, Brazil; 10 mg/kg body weight) and 10% ketamine hydrochloride (10 mg/mL) (Dopalen, Ceva Saúde Animal Ltda, Paulínia, SP, Brazil; 80 mg/kg body weight). The animals were monitored daily for ligature retention and for any adverse clinical signs, such as respiratory distress, lethargy, abnormal posture, inability to access food or water, and wounds. No adverse effects were observed during the experiment.

The animals were euthanized on day T+14 with a lethal dose of 2% xylazine hydrochloride (2 mg/mL) (Rompum, Bayer Animal Health, São Paulo, SP, Brazil) and 10% ketamine hydrochloride (10 mg/mL) (Dopalen, Ceva Saúde Animal Ltda., Paulínia, SP, Brazil). Hemi mandibles and surrounding tissues, as well as segments of the small intestine (duodenum, jejunum, and ileum), were excised for processing.

### 2.4 Micro‐computed tomography (micro‐CT) analysis

Non‐demineralized left hemi‐mandibles were scanned using a micro‐CT system (Skyscan 1172, Bruker, Kontich, Belgium; Supporting Information S1 in the online *Journal of Periodontology*), as described previously.^22^ Linear measurements of alveolar bone level (ABL) were performed using CT‐Analyzer software (version 1.13.5.1+, Bruker, Kontich, Belgium) between the cementoenamel junction (CEJ) and the alveolar bone crest (ABC) at the buccal, lingual, mesial, and distal surfaces of M1, as described previously^27^ (Supporting Information S1). In the furcation area, ABL was assessed as the distance from the roof of the furcation to the inter‐radicular ABC at three sites (mesial, central, and distal). Linear measurements from all seven sites were summed to represent the ABL value for each animal.

For volumetric analysis (CT‐Analyzer, version 1.13.5.1+, Bruker, Kontich, Belgium), a volume of interest (prismatic section) was delineated from the root apices to the roof of the furcation of M1, encompassing the root surfaces.^22^ The volumetric parameters evaluated included: percent bone volume (BV/TV, %), bone surface/volume ratio (BS/BV, 1/mm), trabecular thickness (Tb.Th, mm), trabecular number (Tb.N, 1/mm), trabecular separation (Tb.Sp, mm), and total porosity (percent) (Po(tot, %). Three‐dimensional rendered images of the microtomographic sections were generated using CTVox software (version 3.1.0, Bruker, Kontich, Belgium).

### 2.5 Histopathological analysis of periodontal tissues

The periodontal tissues and M1 were processed for routine histological examination, as described previously.^26^ Serial paraffin sections (4 µm thick) were obtained in the mesiodistal direction, mounted on glass slides, and stained with hematoxylin and eosin (H&E). The periodontal tissues in the furcation area were evaluated based on specific parameters, as described previously^28^ (Supporting Information S1).

### 2.6 Immunohistochemical analysis of periodontal tissues

Immunohistochemical processing was performed using the indirect immunoperoxidase method.^28^ Histological sections of the furcation areas were analyzed under bright‐field illumination using a light microscope (Axiolab, Carl Zeiss, Oberkochen, Germany). Quantitative analysis was performed for TRAP‐positive (tartrate‐resistant acid phosphatase) cells, and semi‐quantitative analysis was conducted for the immunolabeling of TNF‐α, IL‐1β, IL‐10, TGF‐β1, and cytokine‐induced neutrophil chemoattractant 1 (CINC‐1) (Supporting Information S1).

### 2.7 Histomorphometric analysis of the intestine

Small intestine samples were routinely processed and embedded in paraffin for the preparation of serial sections with a thickness of 6 µm, which were stained with H&E. Three histological sections from each portion of the small intestine (duodenum, jejunum, and ileum) displaying villi oriented perpendicularly to the intestinal muscular layer were selected. Images of the histological sections were captured using a high‐resolution digital camera (DFC 310 FX, Leica Microsystems GmbH, Wetzlar, Germany) attached to a trinocular microscope with bright‐field and polarized light (DMLB, Leica Microsystems GmbH, Wetzlar, Germany) and saved to a computer. Using ImageJ software (National Institutes of Health, Bethesda, MD, USA), the following measurements were performed: (i) villus height (VH)—measured as the vertical distance from the tip of the villus to the villus–crypt junction, in 10 villi per histological section; and (ii) crypt depth (CD)—measured as the vertical distance from the crypt–villus junction to the base of the crypt, in 10 crypts per histological section. The VH and CD values for each animal were represented by the mean of the values obtained from the histological section analyses.^26^ Histopathological analysis of intestinal tissues was performed by a certified histologist (Supporting Information S1).

### 2.8 Immunohistochemical analyses of the intestine

Immunohistochemical processing of the intestinal sections was performed as described previously^28^ (Supporting Information S1). Semi‐quantitative analyses were conducted for the immunolabeling of claudin‐1 (CL‐1), occludin (OC), TNF‐α, IL‐1β, IL‐10, and TGF‐β1.

### 2.9 Examiner calibration

Microtomographic and histometric analyses were performed by calibrated examiners, while histopathological and immunohistochemical analyses were conducted by a certified and calibrated histologist. For calibration of each analysis, one‐third of the sample was evaluated twice, with a 48‐hour interval between assessments. The intraclass correlation coefficient (ICC) was used to determine the reproducibility of the evaluations, with values above 90% ensuring examiner calibration. All examiners were blinded to the experimental groups to which each animal belonged. The study coordinator (F.A.C.F.) was aware of the group allocation during the allocation process, experimental procedures, and data interpretation. The study coordinator did not participate in any of the analyses, ensuring that blinding was maintained during all outcome evaluation procedures.

### 2.10 Statistical analysis

Data normality was assessed using the Shapiro–Wilk test. Inter‐ and intra‐group comparisons at different time points were performed using two‐way ANOVA followed by Tukey's post‐hoc test for micro‐CT analyses, intestinal histometry, and TRAP‐positive cell counts (The Jamovi Project, version 2.3.38, Sydney, Australia, 2024). For immunohistochemical analyses of periodontal and intestinal tissues, as well as histopathological evaluations of periodontal tissues, comparisons were performed using the Kruskal–Wallis test followed by the Student–Newman–Keuls post‐hoc test (BioEstat software version 5.3, Mamirauá Institute, AM, Brazil). The significance level adopted for all analyses was set at 5%.

## 3 RESULTS

### 3.1 Micro‐computed tomography (micro‐CT)

**FIGURE 1:**
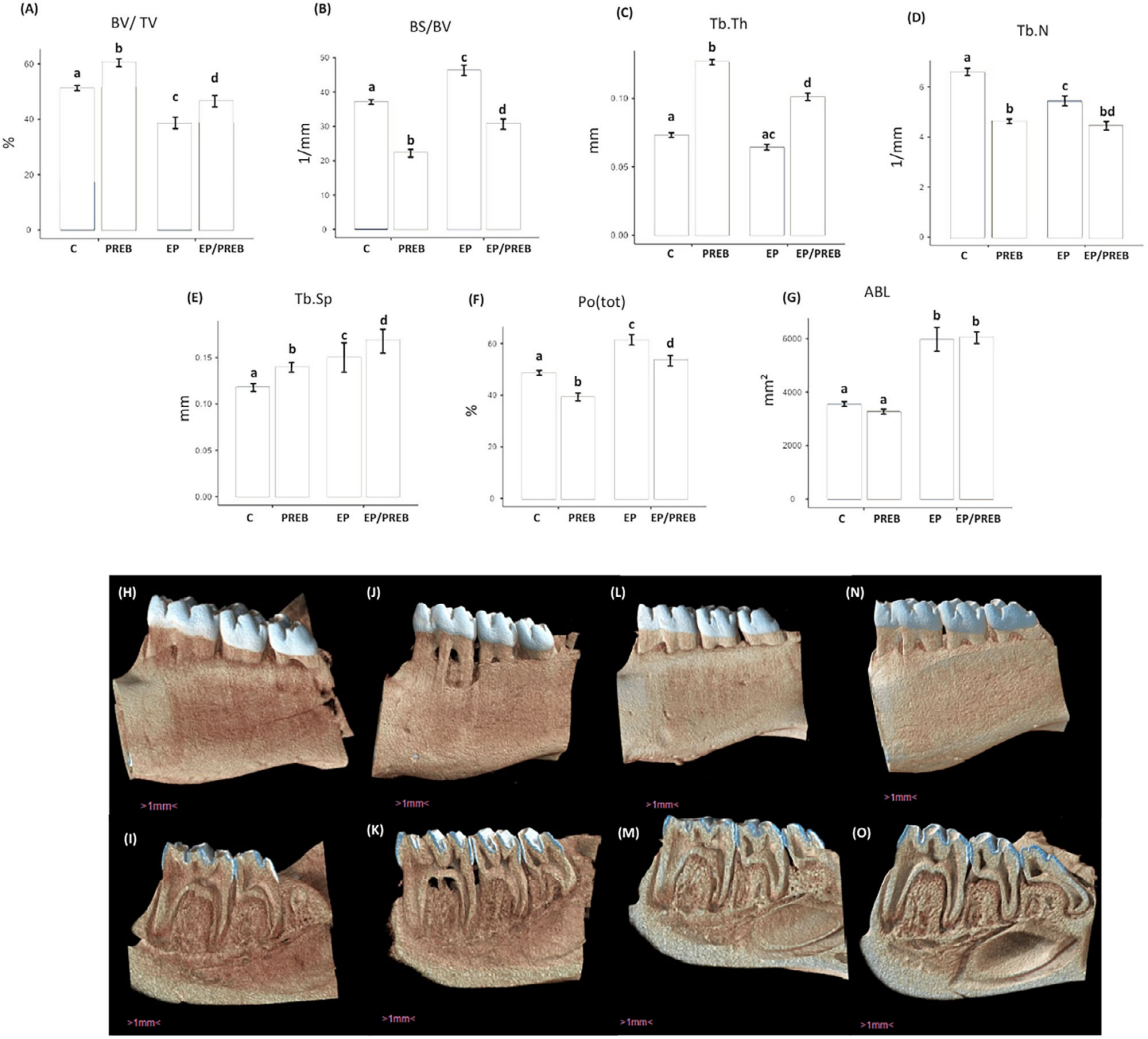
(A–G) Means and standard deviations of the microtomographic analyses of the animals from each experimental group. (A) Percent bone volume (BV/TV, %); (B) Bone surface/volume ratio (BS/BV, 1/mm); (C) Trabecular thickness (Tb.Th, mm); (D) Trabecular number (Tb.N, 1/mm); (E) Trabecular separation (Tb.Sp, mm); (F) Total porosity (percent) (Po[tot], %); (G) Linear measurements expressing the alveolar bone level (ABL, mm^2^) at vestibular, lingual, mesial, distal, and furcation sites. Different letters indicate significant differences between experimental groups (two‐way ANOVA, Tukey, *p <* 0.05). C, control; PREB, prebiotic; EP, experimental periodontitis. (H–O) Representative images of the three‐dimensional renderings of microtomographic sections of the hemi‐mandibles from animals of groups C (H, I), EP (J, K), PREB (L, M), and EP/PREB (N, O). Images of the external vestibular surfaces (H, J, L, N) and sagittal sections showing internal surfaces (I, K, M, O). CTVox (version 3.1.0, Bruker, Kontich, Belgium). Pixel size: 7.96 µm.

Micro‐CT analysis of the non‐demineralized specimens revealed that the EP/PREB group showed higher values of bone volume/total volume (BV/TV), trabecular separation (Tb.Sp), and trabecular thickness (Tb.Th), and lower values of bone surface/bone volume (BS/BV), total porosity (Po[tot]), and trabecular number (Tb.N) compared with the EP group (*p* < 0.05). The PREB group demonstrated higher bone volume/total volume (BV/TV) and trabecular thickness (Tb.Th) values and lower total porosity (Po[tot]) values compared with all other groups (*p <* 0.05). Groups with induced EP (EP and EP/PREB) exhibited significantly greater alveolar bone level than the other groups (*p <* 0.05, Figure 1).

### 3.2 Histopathological analysis of periodontal tissues

No significant differences were observed between the C and PREB groups in the analyzed histopathological parameters (*p >* 0.05). Overall, the EP/PREB group showed lower intensity and extent of the local inflammatory response and a better pattern of gingival connective tissue and alveolar bone tissue organization compared with the EP group (*p <* 0.05, Table S1 in the online *Journal of Periodontology*).

### 3.3 Immunohistochemical analysis of periodontal tissues

**FIGURE 2:**
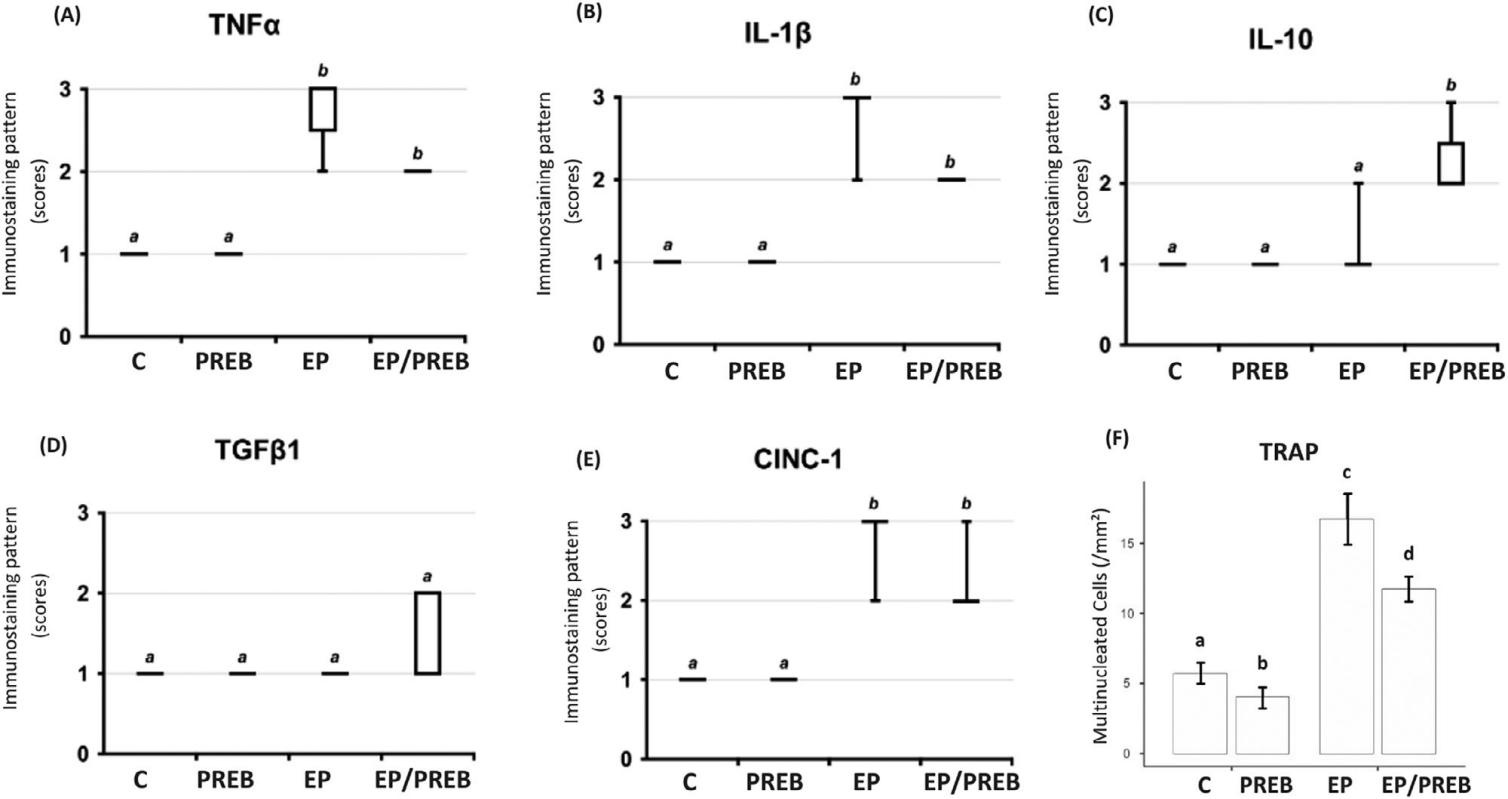
Medians and interquartile ranges of the immunostaining patterns for TNF‐α (A), IL‐1β (B), IL‐10 (C), TGFβ1 (D), CINC‐1 (E) in the periodontal tissue of the animals from each experimental group (Kruskal‐Wallis test, Student‐Newman‐Keuls test, *p <* 0.05). Number of TRAP‐positive cells (F) in the alveolar bone across different experimental groups (two‐way ANOVA, Tukey, *p <* 0.05). Different letters indicate significant differences between experimental groups. C, control; PREB, prebiotic; EP, experimental periodontitis.

**FIGURE 3:**
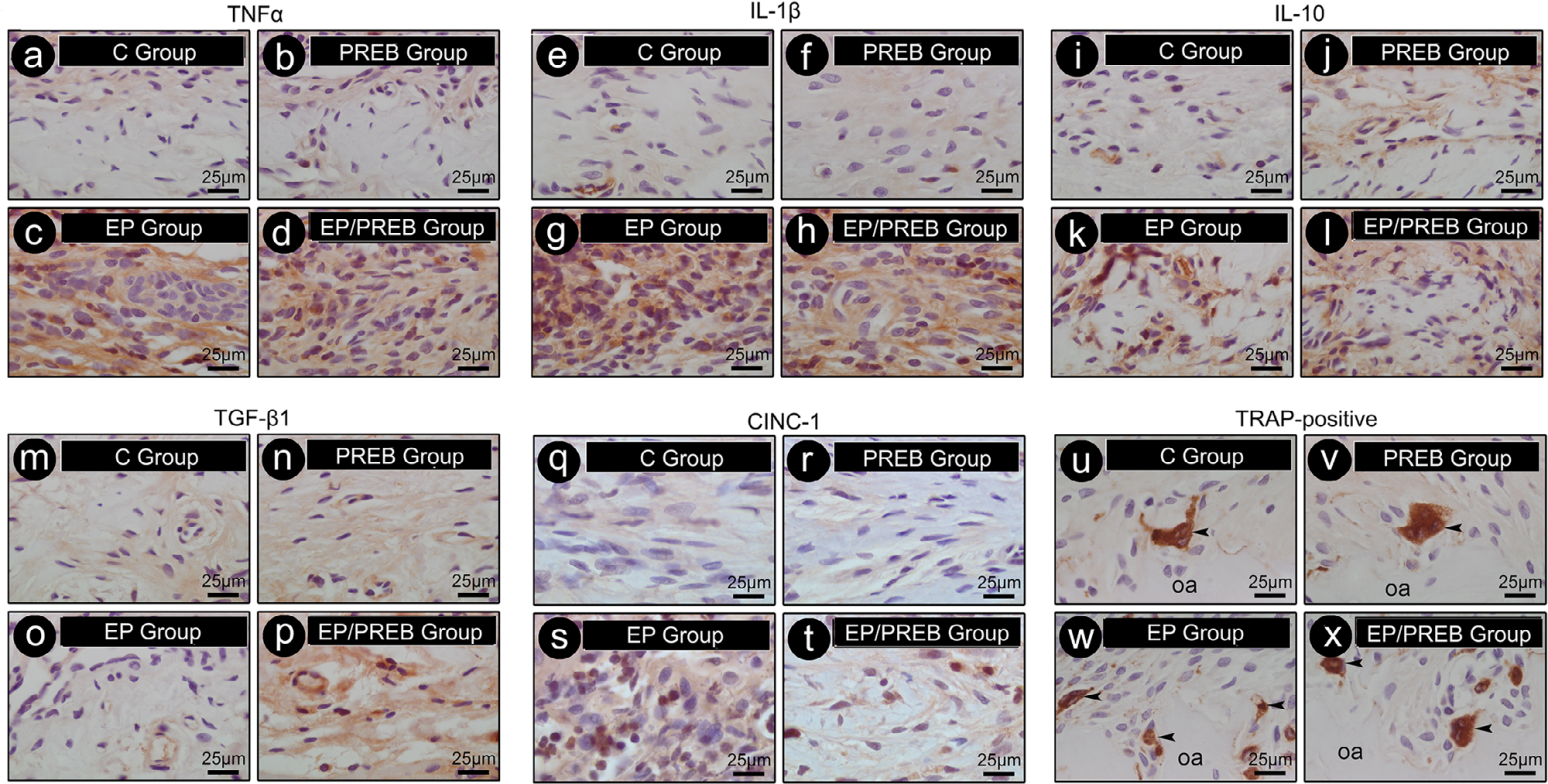
Photomicrographs showing the immunostaining patterns for TNF‐α (A–D), IL‐1β (E–H), IL‐10 (I–L), TGF‐β1 (M–P), and CINC‐1 (Q–T) in the connective periodontal tissue of M1 in groups C (A, E, I, M, Q), PREB (B, F, J, N, R), EP (C, G, K, O, S), and EP/PREB (D, H, L, P, T). Photomicrographs showing the immunostaining patterns for TRAP‐positive cells (U–X) in the alveolar bone of the furcation region of M1 in groups C (U), PREB (V), EP (W), and EP/PREB (X). Counterstaining: Harris Hematoxylin. Original magnification: 1000×. Scale bars: 25 µm.

No significant differences were observed in the immunolabeling patterns for TNF‐α, IL‐1β, and CINC‐1 between the EP and EP/PREB groups (*p >* 0.05). The EP/PREB group exhibited a higher immunolabeling pattern for IL‐10 compared with the other experimental groups (*p <* 0.05). There was no statistically significant difference in the immunolabeling pattern for TGF‐β between the groups (*p >* 0.05). The EP/PREB group had a lower number of TRAP‐positive cells than the EP group (*p <* 0.05, Figures 2 and 3).

### 3.4 Histomorphometric analysis of the intestine

**FIGURE 4:**
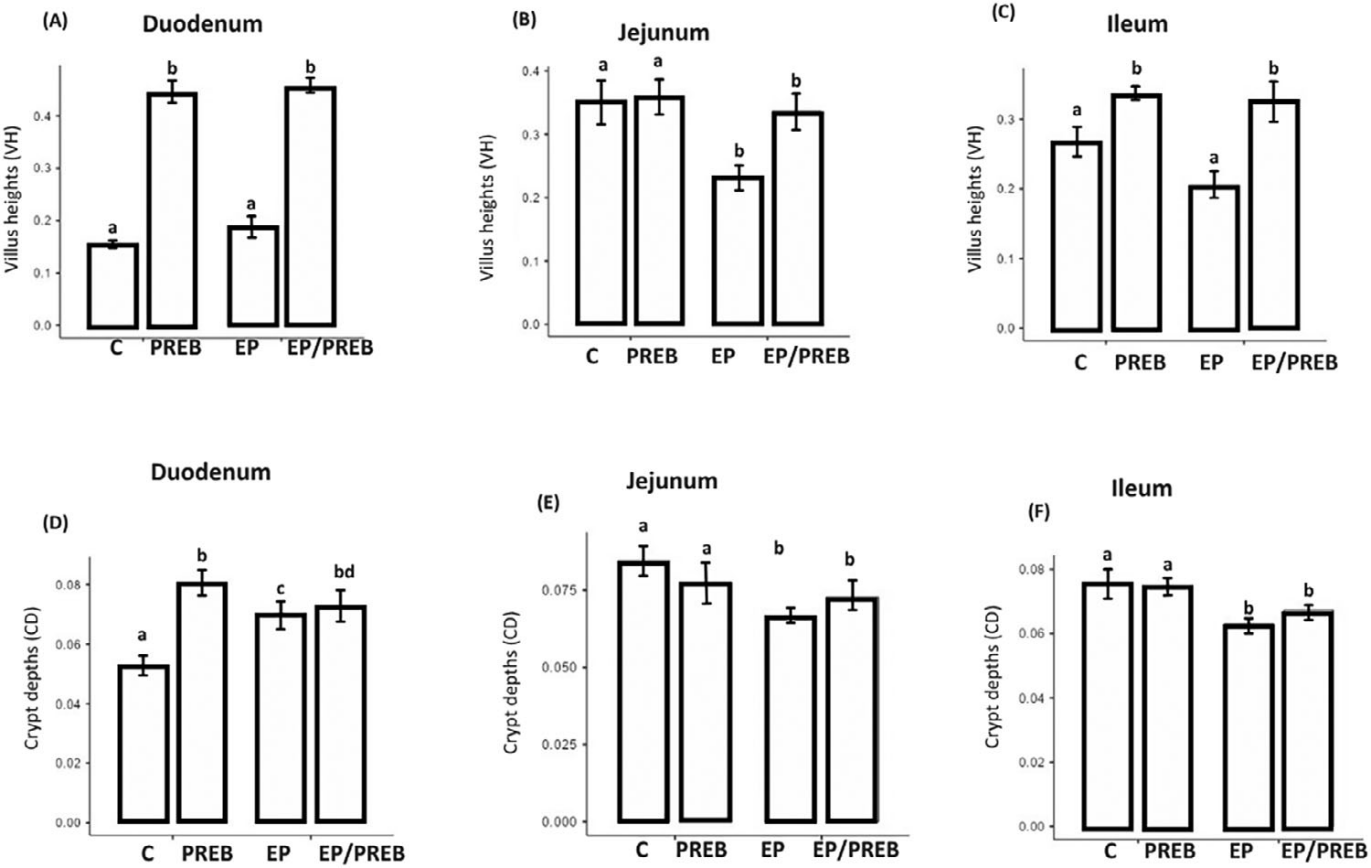
(A–C) Means and standard deviations of villus heights (VH) of the small intestines duodenum (A), jejunum (B), and ileum (C) for C, EP, PREB, and EP/PREB groups, with comparisons between groups. (D–F) Means and standard deviations of crypt depths (CD) of the small intestines duodenum (D), jejunum (E), and ileum (F) for C, EP, PREB, and EP/PREB groups, with comparisons between groups. Different letters indicate significant differences between experimental groups (two‐way ANOVA, Tukey, *p <* 0.05). C, control; PREB, prebiotic; EP, experimental periodontitis.

The EP/PREB group exhibited significantly higher villus height in the duodenum and ileum, and higher crypt depth in the duodenum compared with the EP group (*p <* 0.05). In some intestinal segments, groups consuming PREB showed greater VH (duodenum, ileum) and CD (duodenum) values than groups without PREB consumption (*p <* 0.05). Additionally, the EP group presented lower values than the C group in the jejunum (VH and CD) and ileum (CD) (*p <* 0.05, Figure 4).

Histopathological analysis of the intestines showed intact mucosae in the duodenum, jejunum, and ileum, with patterns consistent with normal histology. No histological differences were observed in the mucosae of the duodenum, jejunum, or ileum when comparing the different groups. The connective tissues and muscle layers appeared intact and normal in all intestinal segments and experimental groups, as did the local vasculature (Figure S1 in the online *Journal of Periodontology*).

### 3.5 Immunohistochemical analyses of the intestine

**FIGURE 5:**
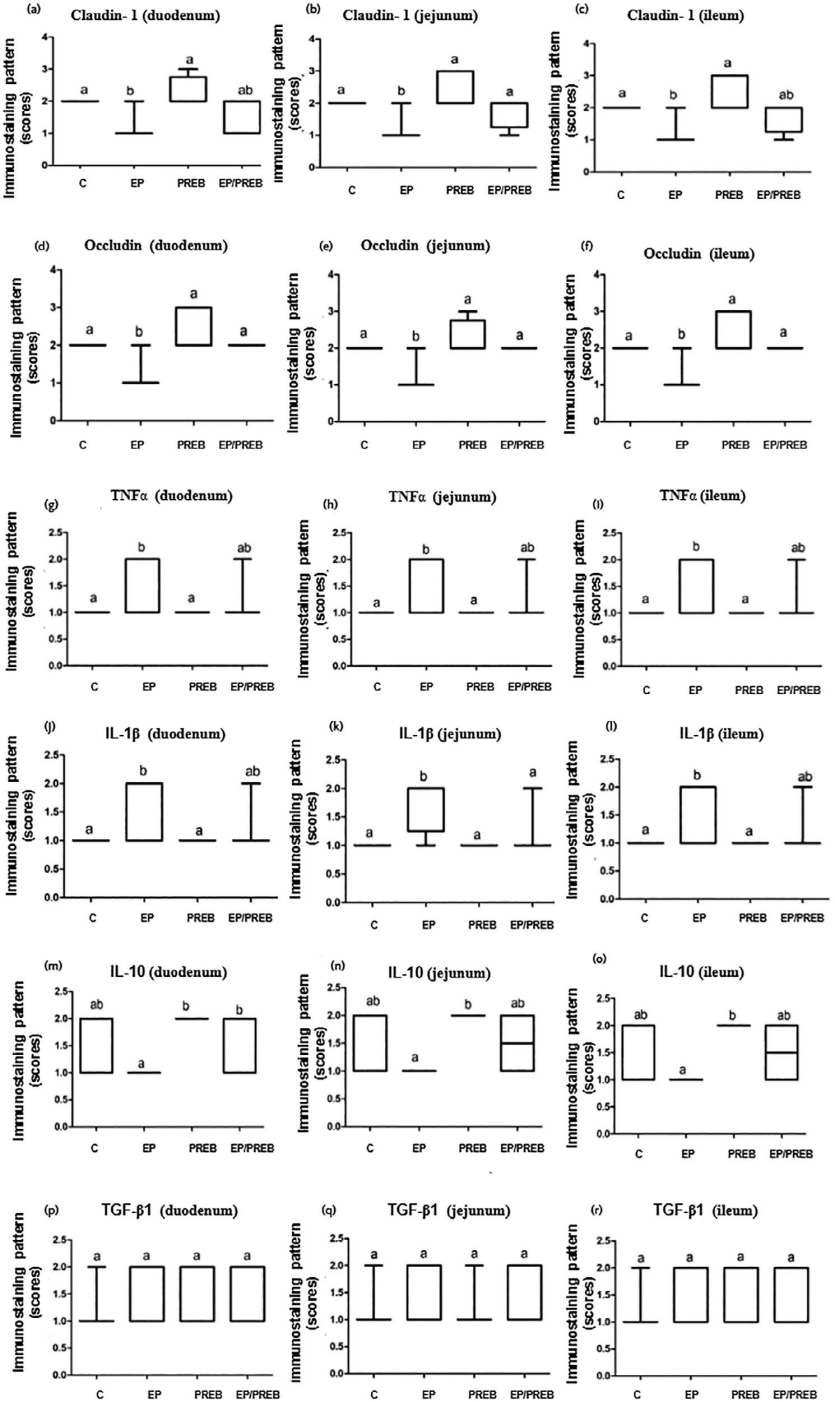
Medians and interquartile ranges of the immunostaining patterns in the small intestines of the animals from each experimental group. Claudin‐1 in duodenum (A), jejunum (B), and ileum (C); occludin in duodenum (D), jejunum (E), and ileum (F); TNF‐α in duodenum (G), jejunum (H), and ileum (I); IL‐1β in duodenum (J), jejunum (K), and ileum (L); IL‐10 in duodenum (M), jejunum (N), and ileum (O); TGFβ1 in duodenum (P), jejunum (Q), and ileum (R). Different letters indicate significant differences between experimental groups (the Kruskal–Wallis test, Student–Newman–Keuls test, *p <* 0.05). C, control; PREB, prebiotic; EP, experimental periodontitis.

**FIGURE 6:**
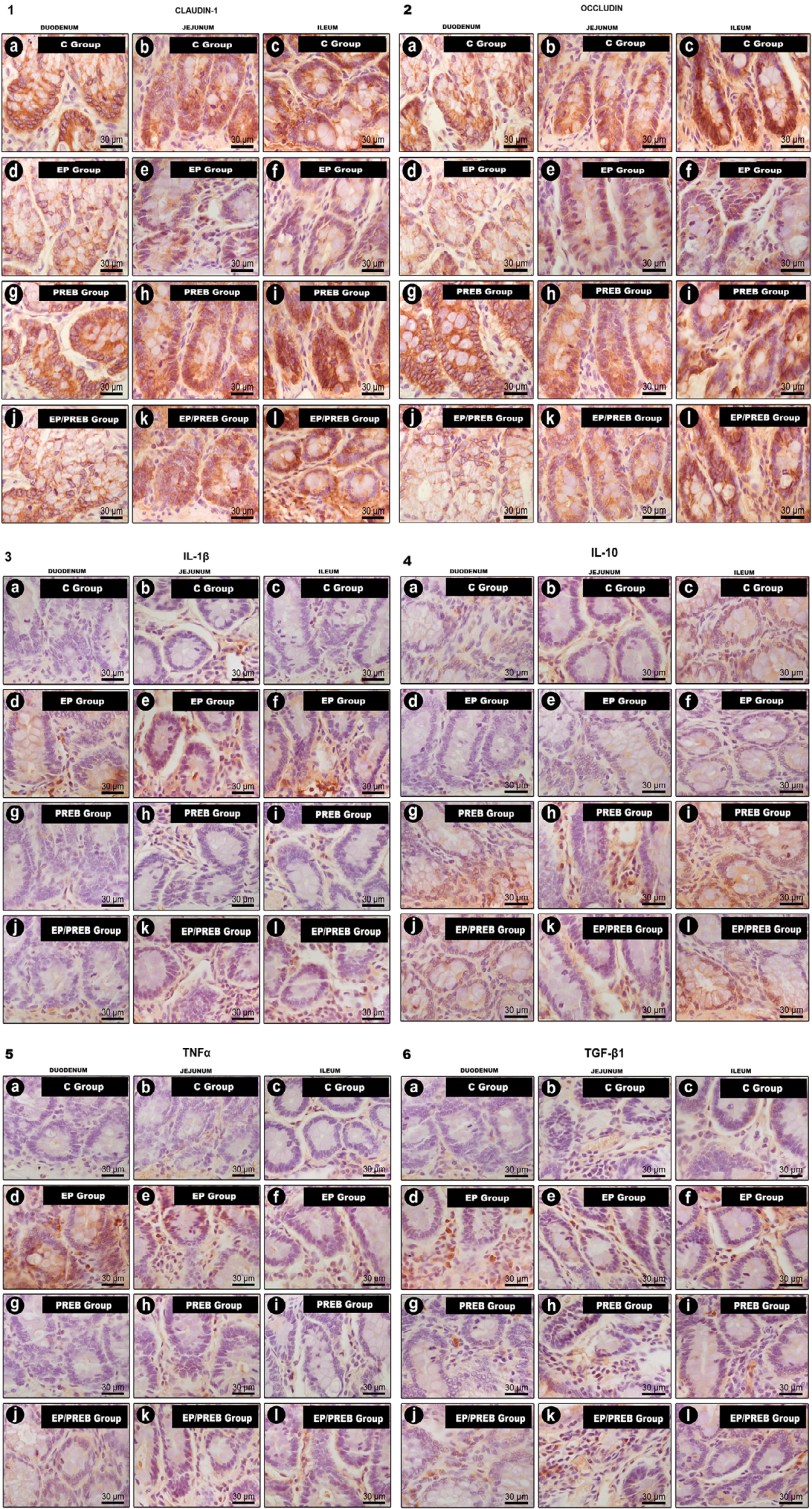
Photomicrographs showing the immunostaining patterns for Claudin‐1 (Frame 1: A–L); Occludin (Frame 2: A–L); IL‐1β (Frame 3: A–L); IL‐10 (Frame 4: A–L); TNF‐α (Frame 5: A–L) and TGF‐β1 (Frame 6: A–L) in the intestinal mucosa of small intestines (duodenum, jejunum, and ileum) of groups C, EP, PREB, and EP/PREB. Counterstaining: Harris Hematoxylin. Original magnification: 1000×. Scale bars: 30 µm.

In the duodenum, jejunum, and ileum, the EP group exhibited a lower immunolabeling pattern for CL‐1 compared with the C and PREB groups, and also a lower pattern for OC than the C, PREB, and EP/PREB groups (*p <* 0.05). In the jejunum, the EP/PREB group showed a higher immunolabeling pattern for CL‐1 compared with the EP group (*p <* 0.05). The EP group demonstrated increased immunolabeling for TNF‐α and IL‐1β compared with the C and PREB groups in the duodenum, jejunum, and ileum (*p <* 0.05). The EP/PREB group showed reduced immunolabeling for IL‐1β compared with the EP group in the jejunum (*p <* 0.05). The EP group exhibited a lower immunolabeling pattern for IL‐10 than the PREB group in all portions of the small intestine (*p <* 0.05). In the duodenum, the EP/PREB group displayed higher IL‐10 immunolabeling compared with the EP group (*p <* 0.05). No statistically significant differences were found in the immunolabeling pattern for TGF‐β1 among the experimental groups in the intestinal sections (*p >* 0.05, Figures 5 and 6).

## 4 DISCUSSION

The effects of some PREBs, such as mannan‐oligosaccharides and β‐glucans, have been investigated in experimental periodontitis.^10^, ^11^ However, this is the first study to evaluate the effects of oral administration of the PREB PDX on the development of EP. When assessing the bone volume in the furcation region of non‐demineralized specimens by micro‐CT, the EP/PREB group showed significantly better results in tridimensional parameters such as bone volume/total volume (BV/TV%), trabecular thickness (Tb.Th), trabecular separation (Tb.Sp), and total porosity (Po[tot]) compared with the EP group. On the other hand, no differences between these groups were observed when assessing the two‐dimensional parameters included in the ABL outcome. It is important to emphasize that periodontitis is a site‐specific disease, potentially affecting some tooth surfaces while sparing others. Furthermore, the more favorable trabecular microarchitecture observed in the EP/PREB group may contribute to a slower progression of periodontitis over time.^29^ A weak trabecular pattern of the alveolar bone has been suggested to be associated with increased susceptibility and a faster progression of periodontitis, highlighting the potential relevance of microarchitectural alterations.^30^ Indeed, the lower expression of TRAP in the bone tissue of the EP/PREB group in comparison with the EP reflects the reduction in osteoclastic activity provided by the PREB. It is also noteworthy that the group without EP that received the PREB (PREB group) showed superiority in some bone volume and microarchitecture parameters (such as bone volume/total volume [BV/TV%], trabecular thickness [Tb.Th], and total porosity [Po{tot}]) compared with the other groups, including the control group (C). Consistent with these findings, a previous study demonstrated that supplementation of pregnant rats with inulin enriched with oligofructose resulted in positive effects on bone mass and vertebral architecture of their offspring in early adulthood, including increased bone volume and trabecular thickness, as well as reduced cortical porosity.^31^

The action of polydextrose on bone tissue observed in this study corroborates previous findings in ovariectomized rats that consumed PDX and showed increased calcium absorption, thereby improving its bioavailability and preventing bone mass loss.^32^ However, unlike the cited study,^32^ the present study included only male rats in order to minimize biological variability associated with hormonal fluctuations during the female estrous cycle.^33^, ^34^ In this context, several other studies also point to mechanisms by which different PREBs favor bone metabolism. Silva et al. (2017) observed that administration of β‐glucan extracted from *Saccharomyces cerevisiae* led to a reduction in alveolar bone loss in both healthy rats with EP and rats with EP and streptozotocin‐induced diabetes mellitus.^10^ Mannan‐oligosaccharides are also PREBs with demonstrated beneficial effects on alveolar bone loss in rats with induced experimental periodontitis.^11^

Modifications in the intestinal microbiota promoted by the administration of probiotics and PREBs may exert osteoimmunomodulatory and osteoprotective effects, involving mechanisms that modulate the immune system and regulate the endocrine system.^35^ Maintenance of a commensal oral microbiota may also be associated with the regulation of bone remodeling processes mediated by osteoclasts and osteoblasts through immunomodulatory activity.^36^ PREBs stimulate SCFAs production, such as butyrate and propionate, by the intestinal microbiota, which promote increased bone mass and prevent bone loss caused by inflammatory processes in osteoporosis by downregulating osteoclast gene expression.^37^ Furthermore, they modulate immunity by increasing regulatory T cells (which inhibit osteoclasts) and blocking TNF receptor‐associated factor 6 (TRAF6) and the nuclear factor of activated T cells—key molecules in bone metabolism signaling.^37^ Thus, as a diet rich in PREBs and probiotics emerges as an innovative therapeutic strategy in the gut–bone axis for the treatment of bone diseases such as osteoporosis,^37^ it is hypothesized that similar benefits may also occur for periodontal bone tissue.

In line with the extensive scientific literature demonstrating that experimental periodontitis can exert systemic effects, including on intestinal tissues,^11^, ^26^, ^38^ the present study observed a reduction in villus height in the jejunum and crypt depth in the ileum of the EP group compared with the control. Indeed, previous studies have reported that animals with EP exhibited intestinal mucosa with morphological alterations in VH and CD, basal lamina degeneration, and neutrophilic infiltration.^26^, ^38^ In this study, analysis of the duodenum revealed increases in VH and CD in animals receiving PREBs (PE/PREB and PREB groups) compared with untreated animals (C and EP groups). Similarly, β‐glucan derived from *Saccharomyces cerevisiae* has been shown to increase crypt depth in diabetic and non‐diabetic animals treated with the PREB.^10^

In the immunohistochemical analyses, the EP/PREB group showed lower immunolabeling for IL‐1β in the jejunum and higher immunolabeling for IL‐10 in the duodenum and periodontal tissues compared with the EP group. Elevated levels of IL‐1β contribute to intestinal inflammation in inflammatory bowel diseases (IBD)^39^ and are also associated with the severity, progression, and recurrence of periodontitis, as well as tooth loss.^40^ Considering also that IL‐10 plays an important role in regulating the inflammatory response and maintaining immune balance in the intestine,^41^ and inhibits bone resorption in various tissues, including periodontal tissues,^42^ these results demonstrate an important immunomodulatory effect of PDX. Indeed, previous studies have demonstrated the immunomodulatory potential of different PREBs.^43^, ^44^, ^45^ Fructo‐oligosaccharide increased IL‐10 expression in dendritic cells of the rectal mucosa of patients with Crohn's disease.^44^ Administration of β‐glucans from *Saccharomyces cerevisiae* was able to reduce serum levels of IL‐1β^45^ and TNF‐α^10^, ^45^ in rats with periodontitis and diabetes mellitus. Mannanoligosaccharides reduced periodontal destruction and the expression of TNF‐α and IL‐1β and increased TGF‐β immunolabeling in periodontal tissues of rats with periodontitis.^11^ Specifically regarding PDX, it reduced serum levels of IL‐1β and IL‐6 and increased serum levels of IL‐10 in mice fed a high‐fat diet.^43^ In swine, supplementation with PDX at doses up to 8.5 g/L decreased intestinal gene expression of TNF‐α, IL‐8, and IL‐1β, and doses of 1.7 and 17 g/L increased intestinal levels of IL‐10.^46^ The immunomodulatory effects of PDX were also observed in the histopathological analyses of periodontal tissues of the animals in this study, where the EP/PREB group showed lower intensity and extent of local inflammatory response and a better pattern of gingival connective tissue and alveolar bone structure compared with the EP group. Similar effects, with a reduction of inflammation and destruction of periodontal tissues, were observed with the use of the PREBs mannanoligosaccharides and β‐glucan derived from *Saccharomyces cerevisiae*.^10^, ^11^

Claudins and occludins are integral membrane proteins that structurally contribute to the junctions of epithelial cells composing the intestinal barrier, ensuring a strict and controlled separation between the intestinal lumen contents and the internal environment of the organism.^47^ When the integrity of the intestinal barrier is compromised, its permeability may increase, allowing microorganisms and their products, such as toxins and lipopolysaccharides, to translocate into the bloodstream and cause endotoxemia and systemic inflammation.^47^ In this study, while animals with EP exhibited lower intestinal expression of CL‐1 and OC than animals without EP, animals with EP treated with PDX showed CL‐1 expression in the jejunum and OC expression in all intestinal portions similar to those of the control (C) and PREB groups. These results suggest that supplementation with PDX may minimize the damage caused by EP to the transmembrane proteins of the intestinal barrier, potentially mitigating the systemic repercussions of EP. In previous studies, xylo‐oligosaccharide was shown to increase CL‐1 expression in the colon of mice,^48^ pectin improved intestinal barrier function and increased OC expression in pigs,^49^ and inulin was able to restore the integrity and function of the intestinal barrier, increasing CL‐1 and OC expression in rats with hepatic steatosis.^50^

It is important to emphasize that, just like probiotics, PREBs also have unique and specific mechanisms of action that are dependent on the type of fiber, which directly impacts the suitability of their use for different preventive and therapeutic health approaches. For example, pectin is capable of reducing alkaline phosphatase activity and increasing bile acid concentrations,^49^ which have immunomodulatory and metabolic properties,^51^ while β‐glucan promotes pancreatic beta cell function, suggesting an adjuvant role in glucose metabolism and hyperglycemia control.^10^ Polydextrose, on the other hand, has demonstrated mechanisms of action including favorable modulation of the intestinal microbiota, such as stimulating the growth of bifidobacteria and lactobacilli, increasing concentrations of propionic and lactic acids, which contribute to maintaining intestinal pH,^16^, ^46^ and balancing lipid metabolism,^16^, ^19^ in addition to those observed in the present study and discussed above. The production of metabolites such as SCFAs from PREBs, including PDX, represents an important effect of these substances.^18^ The assessment of SCFA levels, which was not performed in the present study, could contribute to a better understanding of the mechanisms of action of PDX, especially regarding its impacts on bone quality.

Another important aspect to consider in the use of PREBs is the daily dosage and duration of administration, which can impact the effects of PREBs on immunomodulation and bone metabolism. The PDX dosage used in this study, over a period of 44 days, demonstrated benefits in the EP model. This dosage choice was based on the absence of toxic effects, beneficial results on the intestinal mucosa, increased SCFA production, improved calcium absorption, and bone mineralization observed in other experimental models, with doses ranging from 1.5 to 3 g/day.^23^, ^24^, ^25^ However, as extensively explored in other studies with PREBs under various health conditions,^52^, ^53^ including periodontitis,^10^, ^11^, ^54^ variations in PDX dosage and duration of administration may have different local and systemic impacts than those observed in this study. Therefore, it is important to investigate different dosages, durations, and routes of PDX administration, also using phylogenetically more advanced models, to better clarify the effects of this therapy and its potential clinical application. Furthermore, another limitation of the present study is the absence of microbiological analyses as well as the evaluation of barrier‐related markers in periodontal tissues, which are necessary to better understand the potential effects of PDX on oral and intestinal tissues and, consequently, its local and systemic outcomes.

## 5 CONCLUSION

Within the limitations of this study, it can be concluded that the administration of the PREB PDX promoted a protective effect against alveolar bone resorption, possibly through the favorable modulation of the oral and intestinal immune‐inflammatory response in rats with experimental periodontitis.

## AUTHOR CONTRIBUTIONS

All authors have made substantial contributions to the conception and design of the study. **Raquel de S. F. Nassar**: contributed to the experimental procedures, data acquisition, analysis and interpretation, and drafting of the manuscript. **Marcella C. Ribeiro** and **Ana Carolina P. Hernandes**: contributed to the experimental procedures, data acquisition, and critical revision of the manuscript. **Laura Z. Gianduzzo** and **Vanessa de P. S. Pereira**: contributed to the experimental procedures, data acquisition and analysis, and critical revision of the manuscript. **Sérgio L. S. Salvador**, **Edilson Ervolino**, and **Michel R. Messora**: were involved in data analysis and interpretation, as well as the critical revision of the manuscript. **Débora S. F. Sávio**: contributed to data interpretation and critical revision of the manuscript. **Flávia A. C. Furlaneto**: contributed to the study's conception and design, data analysis and interpretation, manuscript drafting, and critical revision. All authors approved the final version of the manuscript for publication.

## CONFLICT OF INTEREST STATEMENT

The authors report no conflicts of interest related to this study.

## Supporting information



Supporting Information

Supporting Information

Supporting Information

## Data Availability

The data that support the findings of this study are available from the corresponding author upon reasonable request.
