# Supplementary figures and images for: Immunological and periodontal benefits of prebiotic polydextrose in rats with induced periodontitis
Source: J Periodontol. 2026 Feb 19;97(7):1529–42. doi: 10.1002/jper.70095 (PMC13380353; doi:10.1002/jper.70095)

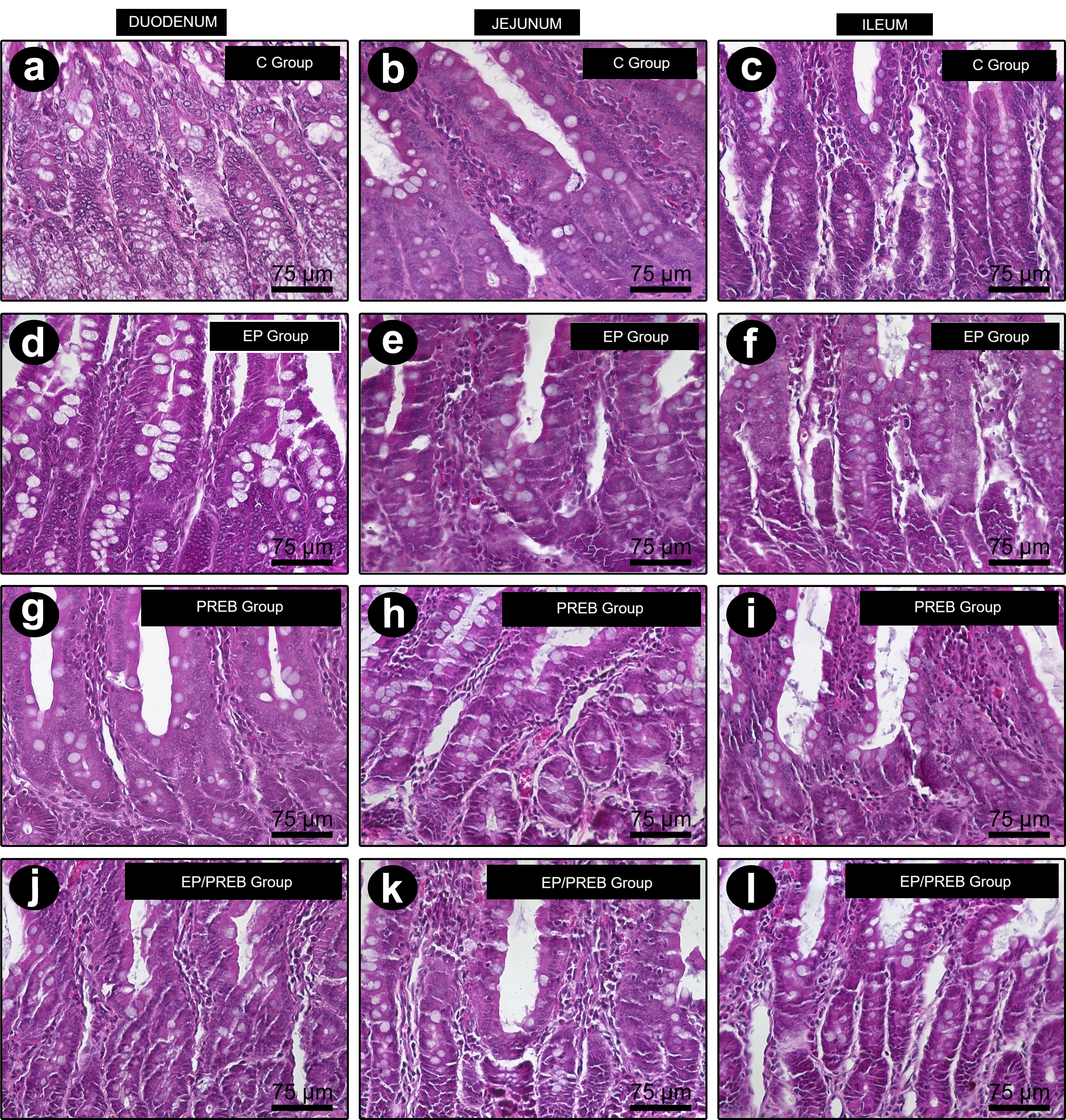

Supplement: Supplementary file 1 — Supporting Information [file JPER-97-1529-s002.png]

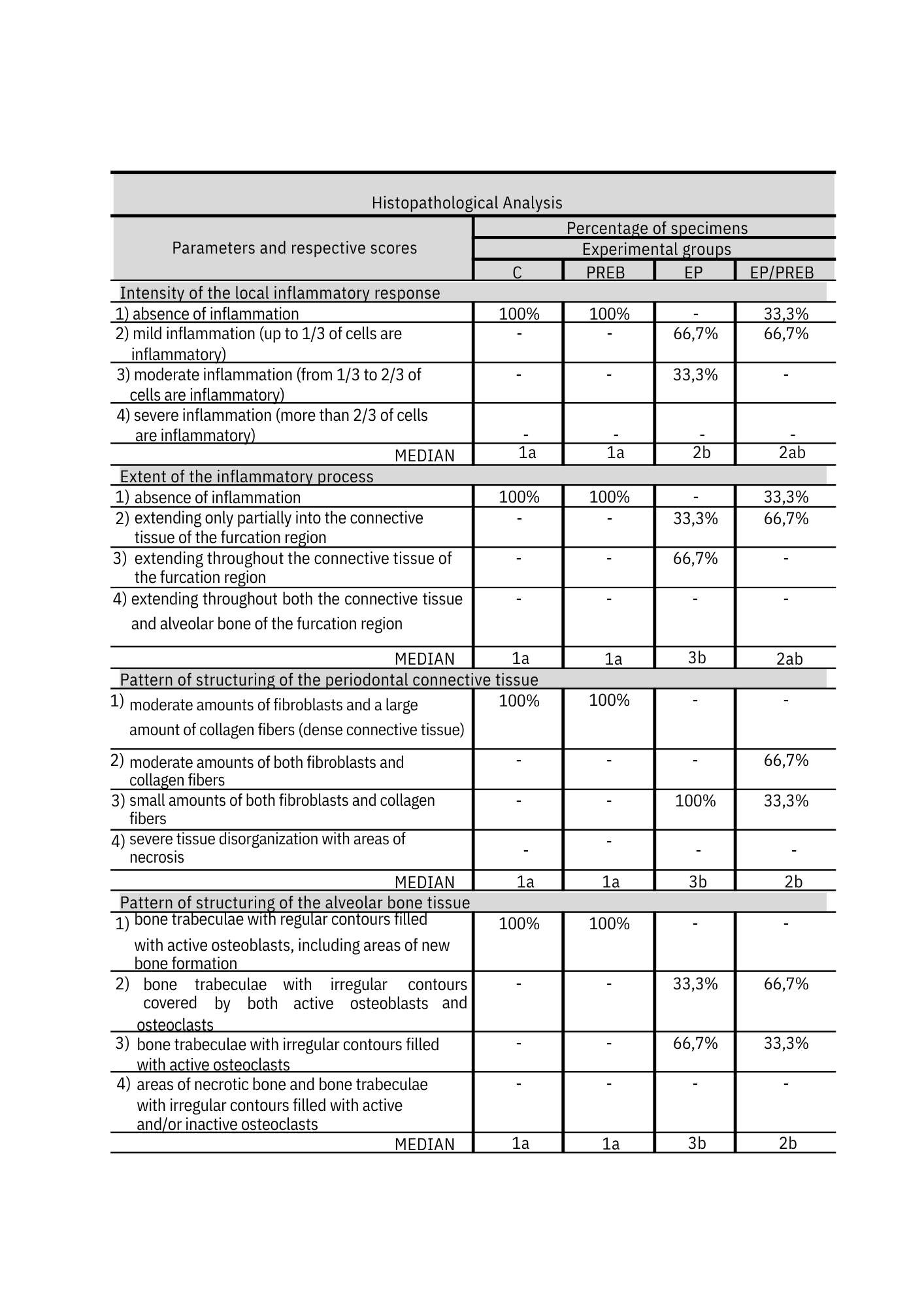

Supplement: Supplementary file 3 — Supporting Information [file JPER-97-1529-s001.png]
