# Supplementary material for: Immunological and periodontal benefits of prebiotic polydextrose in rats with induced periodontitis
Source: J Periodontol. 2026 Feb 19;97(7):1529–42. doi: 10.1002/jper.70095 (PMC13380353; doi:10.1002/jper.70095)
Supplement: Supplementary file 2 — Supporting Information [file JPER-97-1529-s003.docx]

**SUPPORTING INFORMATION S1**

**METHODS**

### **Micro-Computed Tomography (Micro-CT) Analysis**

The X-ray generator was operated at an acceleration voltage of 60 kV, with a beam current of 165 µA and an exposure time of 490 ms per projection. Images were acquired with a voxel size of 6×6×6 µm. Using appropriate software (Data Viewer®, version 1.5.0, Bruker, Kontich, Belgium), the generated three-dimensional models were rotated into a standardized analysis position according to the following criteria: (1) in the transaxial plane, the mandibular first molar (M1) was aligned vertically along its long axis; (2) in the sagittal plane, the occlusal surface of M1 was oriented horizontally; and (3) in the coronal plane, the mandibular bone was aligned vertically, with the mesial root of M1 positioned at the uppermost point of the image^1^.

### **Histopathological Analysis of Periodontal Tissues**

For the histopathological analysis, the periodontal tissues in the furcation region were evaluated based on the following histological parameters: nature and degree of inflammation; extent of the inflammatory process; presence and extent of tissue necrosis; presence, extent, and nature of bone, cementum, and dentin resorption; condition of blood vessels; structural pattern of the extracellular matrix of the periodontal tissues; and cellularity pattern of the periodontal tissues, as previously described^2^.

### **Immunohistochemical Analysis of Periodontal Tissues**

Immunohistochemical processing was performed using the indirect immunoperoxidase method (Furlaneto et al., 2014). The histologic sections were deparaffinized and rehydrated through a descending ethanol series. Antigen retrieval was performed by immersing the slides in 10mM citrate buffer, pH 6.0 (Spring Bioscience, CA, USA) and heating them in a pressurized chamber (Decloaking Chamber®, Biocare Medical, CA, USA) at 95ºC for 20 minutes. At the end of each stage of the immunohistochemical reaction, the slides were washed in 0.1 M PBS, pH 7.4. Subsequently, the histological sections were immersed in 3% hydrogen peroxide for 1 hour and then 1% bovine serum albumin for 12 hours to block the endogenous peroxidase and non-specific sites, respectively. Histological sections containing samples from each experimental group were divided into six batches, and each batch was incubated with one of the following primary antibodies for 24 hours: anti-TNF-α raised in goat against rat (Abcam Laboratories, Cambridge, UK), anti-IL-1β raised in rabbit against rat (Abcam Laboratories, Cambridge, UK), anti-IL-10 raised in goat against rat (Abcam Laboratories, Cambridge, UK), anti-TGF-β1 raised in rabbit against rat (Abcam Laboratories, Cambridge, UK), anti-CINC1 raised in goat against rat (Biorbyt Explore Bioreagents, NC, USA), and anti-TRAP raised in goat against rat (Santa Cruz Biotechnology, Dallas, TX, USA). The sections were then incubated with a biotinylated secondary antibody for 2 hours and subsequently treated with a streptavidin–horseradish peroxidase conjugate for 1 hour (Santa Cruz Biotechnology, Dallas, TX, USA). The reaction was developed using chromogen 3,3'-diaminobenzidine (DAB Chromogen Kit, Dako Laboratories, CA, USA) and counterstained with hematoxylin.

The histological sections were analyzed under bright-field illumination using a light microscope (Axiolab, Carl Zeiss, Oberkochen, Germany) by a certified histologist. Quantitative analyses of TRAP-positive cells and semi-quantitative analyses for immunostaining of TNF-α, IL-1β, IL-10, TGF-β1, and CINC-1 were performed. For TNF-α, IL-1β, IL-10, TGF-β1, and CINC-1, immunostaining was defined as a brown coloration confined to the cytoplasmic compartment of immunoreactive (IR) cells and the extracellular matrix. For each specimen, one histological section from the furcation region was analyzed at 200× magnification. A semi-quantitative analysis was performed, and the scoring criteria were as follows: score 0 – absence of immunostaining [no IR cells and no labeling in the extracellular matrix (ECM)]; score 1 – low immunostaining pattern (≈ 1/4 of IR cells and weak ECM staining); score 2 – moderate immunostaining pattern (≈ 1/2 of IR cells and moderate ECM staining); score 3 – high immunostaining pattern (≈ 3/4 of IR cells and moderate ECM staining). The immunohistochemical technique used for the detection of TNFα, IL-1β, IL-10, TGFβ1, and CINC-1 demonstrated high specificity for these proteins, as evidenced by the complete absence of staining in the negative control of the immunohistochemical reaction (specimens underwent the same procedures with the omission of the primary antibody).

For TRAP, immunostaining was defined as a brown coloration exclusively confined to the cytoplasmic compartment of IR cells. In each specimen, a 1 mm² area in the furcation region was analyzed at 200× magnification. A square with 1000 µm sides was positioned at the center of the interradicular septum. The coronal boundary of the square was the alveolar bone crest, from which it extended 1000 µm apically. Within this region, TRAP-positive multinucleated cells were counted. The number of TRAP-positive cells was expressed as the number of cells per mm² in each experimental group.

**Histomorphometric Analysis of the Intestine**

Histopathological analysis was conducted across all tissue layers comprising the wall of the various segments of the small intestine, with particular focus on the intestinal mucosa. The following parameters were assessed: cellularity pattern and structural organization of the epithelial layer; cellularity and architecture of the intestinal glands; structural and cellular features of the connective and muscular tissues; presence or absence of inflammation; if present, the nature and intensity of the local inflammatory response; the extent of the inflammatory infiltrate; and the condition of the local vasculature.

**Intestinal Immunohistochemical Analyses**

For the immunohistochemical analyses of the intestinal tissues, the histological sections were deparaffinized in xylene and rehydrated through a descending ethanol series. Antigen retrieval was performed by immersing the slides in 10 mM citrate buffer, pH 6.0 (Spring Bioscience, CA, USA), and heating them in a pressurized chamber (Decloaking Chamber®, Biocare Medical, CA, USA) at 95°C. At the end of each step of the immunohistochemical reaction, the slides were washed in 0.1 M PBS, pH 7.4. Subsequently, the histological sections were immersed in a solution of 3% hydrogen peroxide in PBS for 1 hour, followed by immersion in a solution of 4% skim milk in PBS for an additional hour to block endogenous peroxidase and biotin, respectively. Nonspecific binding sites were blocked with a solution containing 1.5% bovine serum albumin in PBS supplemented with 0.05% Triton® X-100 (Sigma-Aldrich®, Merck KGaA, Darmstadt, Germany) for 12 hours. Histological slides containing intestinal tissue sections from each experimental group were divided into six batches, and each batch was incubated for 24 hours with one of the following primary antibodies: anti-claudin-1, anti-occludin, anti-TNFα, anti-IL-1β, anti-IL-10, and anti-TGFβ1 (Abcam Laboratories, Cambridge, UK). Subsequently, the sections were incubated with a biotinylated secondary antibody (©Vector Laboratories, Newark, CA, USA) for 2 hours, followed by treatment with horseradish peroxidase-conjugated streptavidin (©Vector Laboratories, Newark, CA, USA) for another 2 hours. Immunoreactivity was visualized using 3,3’-diaminobenzidine (DAB) as the chromogen (©Vector Laboratories, Newark, CA, USA). The sections were counterstained with Harris hematoxylin, dehydrated in ethanol, cleared in xylene, and coverslipped with mounting medium and glass coverslips. As a negative control, specimens underwent the same procedures with the omission of the primary antibody. All steps of the immunohistochemical procedure were based on the protocol described by Furlaneto et al. (2014). A semi-quantitative analysis was performed, and the scoring criteria were as follows: score 0 – absence of immunostaining; score 1 – low immunostaining pattern; score 2 – moderate immunostaining pattern; score 3 – high immunostaining pattern.

**REFERENCES**

1. MESSORA, MR. et al. Favourable effects of Bacillus subtilis and Bacillus licheniformis on experimental periodontitis in rats. Arch Oral Biol. 2016 Jun;66:108-19. doi: 10.1016/j.archoralbio.2016.02.014. Epub 2016 Feb 26. PMID: 26945169.
2. FURLANETO, F.A, et al. Effects of locally administered tiludronic acid on experimental periodontitis in rats. Journal of Periodontology 2014 Sep;85(9):1291-301. doi: 10.1902/jop.2014.130581. Epub 2014 Feb 6. PMID: 24502611.
